# Physical and Psychological Predictors for Persistent and Recurrent Non‐Specific Neck Pain: A Systematic Review

**DOI:** 10.1002/ejp.70168

**Published:** 2025-11-12

**Authors:** Cho Wai Geoffrey Yu, Kanya Wongwitwichote, Michael Mansfield, Janet A. Deane, Valter Devecchi, Deborah Falla

**Affiliations:** ^1^ University of Birmingham, Centre of Precision Rehabilitation for Spinal Pain (CPR Spine), School of Sport, Exercise and Rehabilitation Sciences, College of Life and Environmental Sciences Birmingham UK

## Abstract

**Background:**

Patients with non‐specific neck pain often develop persistent or recurrent pain and associated disability. This review investigated which physical and psychological factors predict persistent and recurrent neck pain and disability.

**Databases and Data Treatment:**

Five databases were searched from inception to January 31, 2025. After data extraction, the Quality in Prognosis Studies tool (QUIPS) was used for risk of bias assessment and the Grading of Recommendations Assessment, Development and Evaluation (GRADE) tool was used to assess the certainty of evidence for each factor's predictive capability for neck disability, pain intensity and incidence.

**Results:**

Six prospective cohort studies were selected. Three studies were rated with high overall risk of bias and three with moderate overall risk of bias. Consistent findings supported that high pain catastrophizing and psychological distress predict persistent and recurrent pain, disability and incidence with low to moderate certainty of evidence. There was inconsistency of results or limited studies reporting association between neck flexion strength, cold pain threshold tested over the tibialis anterior, pressure pain threshold tested over the neck or tibialis anterior, conditioned pain modulation, neck extensor endurance, and temporal summation with disability. In addition, there was low certainty of evidence which showed no relationship between cervical range of motion, cold pain threshold tested over the neck and neck extensor strength with disability.

**Conclusions:**

Pain catastrophizing and psychological distress were identified as predictors of persistent and recurrent neck pain, disability and incidence. Further studies are needed to confirm findings of an association between physical factors with future neck pain and disability.

**Significance Statement:**

This review provides evidence to support the addition of early assessment of pain catastrophizing and psychological distress to identify patients that are more susceptible to persistent and recurrent neck pain. When warranted, psychological interventions targeting maladaptive beliefs, pain‐related anxiety and catastrophizing may be required to minimize persistent and recurrent neck pain and disability.

## 1 Introduction

Persistence and recurrence of neck pain are common. Persistence refers to neck pain that lasts at least 3 months (Treede et al. 2019), whereas recurrent neck pain has been defined as the experience of episodic pain which lasts for at least 24 h occurring more than once per year with each episode separated by at least 1 month of an asymptomatic period (Stanton et al. 2009). Up to 57% of patients with neck pain experience persistent neck disability for at least 1 year after the initial onset (Vasseljen et al. 2013) and 23%–48% of patients experience a recurrent episode within the first year after onset (Côté et al. 2004; Hill et al. 2004).

Prevention of the persistence and recurrence of neck pain is a challenging task (Cohen 2015) and the development of a predictive model for neck pain is considered a research priority for optimising neck pain management (Cohen 2015; Foster et al. 2009; Silva et al. 2019). However, a systematic review of the literature has not been conducted to summarise predictors for recurrent neck pain. For the prediction of persistent neck pain, a limitation of available reviews (Jahre et al. 2020; McLean et al. 2010; Verwoerd et al. 2019) is the tendency to focus on psychological and demographic features only, with limited reporting on physical factors such as neuromuscular function, sensorimotor control, and sensory alterations which are commonly associated with neck pain (Alsultan et al. 2019; Anarte‐Lazo et al. 2024; Devecchi et al. 2022; Schomacher and Falla 2013; Treleaven 2024; Tsang et al. 2014; Unal et al. 2024; Xie et al. 2021). Interestingly, some of these physical changes have been observed to be present in people with recurrent neck pain during a period of remission (Alalawi et al. 2022; Devecchi, Rushton, et al. 2021). Moreover, a predictive study on recurrent neck pain and disability reported that a higher incidence of episodic pain within the last 12 months along with impaired cervical flexion strength were predictive of higher neck disability at a 6‐month follow‐up (Alalawi et al. 2022). Apart from physical factors, psychological features such as reduced quality of life and kinesiophobia have commonly been found in patients with persistent (Sterling and Chadwick 2010) and recurrent neck pain, even during an asymptomatic period (Devecchi, Falla, et al. 2021). It is important to identify modifiable psychological and physical predictors such that targeted intervention can be applied to minimise recurrent and persistent pain and disability.

This systematic review synthesises evidence regarding the predictive capability of physical and psychological factors for persistent and recurrent pain and disability in people with non‐specific neck pain (NSNP), the most prevalent subtype of neck pain (Blanpied et al. 2017). The objectives are to: (1) identify predictors of persistent and recurrent pain and disability, and (2) evaluate their predictive strength. While it is appreciated that social factors can contribute to pain persistence and recurrence, the specific focus of this review is on modifiable physical and psychological factors for which interventions can be implemented to minimise persistent and recurrent pain and disability.

## 2 Methods

This systematic review followed a published protocol (Yu et al. 2025) which was prospectively registered on the International Prospective Register of Systematic Reviews (PROSPERO; CRD42024596844). The reporting style of the results from this systematic review followed the Preferred Reporting Items for Systematic Reviews and Meta‐Analyses (PRISMA) 2020 statement (Page et al. 2021) and guide to systematic reviews for prognostic factor studies suggested by Riley et al. (Riley et al. 2019).

### 2.1 Eligibility Criteria

The PICOTS framework (Riley et al. 2019) (P—population, I–index predictors, C–comparative predictors, O–Outcomes, T–Timing and S–Setting) was used to define the eligibility criteria for including studies.

*Population*: Adults (≥ 18 years) with persistent or recurrent NSNP were selected as the target population. NSNP is defined as non‐traumatic neck pain where no pathoanatomical origin is identified (Hush et al. 2011). As mentioned above, persistence is defined as pain which has sustained for a minimum of 3 months (Treede et al. 2019). Recurrence is defined as the presence of more than one episode of neck pain which lasted at least 24 h with a separation of at least 30 days of an asymptomatic period between episodes; this definition is modified from the classification of recurrent low back pain developed from a previous systematic review (Stanton et al. 2009).

*Predictors of neck pain persistence and recurrence*: Objective physical measurements including but not limited to neck kinematics, motor output, muscle activity, sensorimotor control and sensory function were defined as the physical predictors. Psychological predictors included common self‐reported psychological features comprising but not limited to the Tampa Scale for Kinesiophobia (TSK) (Cleland et al. 2008), European Quality of life– 5 Dimensions (EQ‐5D) (Brooks 1996), Pain Coping Inventory (PCI) (Kraaimaat and Evers 2003), and Pain Self‐Efficacy Questionnaire (PSEQ) (Chiarotto et al. 2018).

*Outcomes*: The primary outcome of interest was the Neck Disability Index (NDI) (Vernon and Mior 1991). Other outcomes reported in selected studies reflecting the intensity, frequency and incidence of neck pain were investigated in the review and were considered as secondary outcomes which consisted of pain intensity measured using the Numeric Pain Rating Scale (NPRS) or Visual Analog Scale (VAS) scores during an episode of persistent pain or the average pain score within a follow‐up period of recurrent neck pain (Devecchi, Falla, et al. 2021). The frequency of recurrence of neck pain was represented by the number of days with neck pain or the number of neck pain episodes over a particular period (Devecchi, Falla, et al. 2021).

*Timing*: The outcomes should have been assessed as early as a 3‐month follow‐up for persistent neck pain and a 1‐month follow‐up for recurrent neck pain.

*Setting*: This review included physical and psychological predictors that were evaluated in either primary care settings or secondary care settings for the management of neck pain.

*Exclusion criteria*: Case–control studies or case reports were excluded. In addition, cohorts with an active intervention were also excluded to minimize heterogeneity as different patients may respond differently to various treatments leading to confounding effects on the outcome for pain persistence and recurrence.

### 2.2 Search Strategy

The search was conducted from inception to January 31, 2025. The following databases were searched: PubMed, MEDLINE (OVID), EMBASE (OVID), Cumulative Index to Nursing and Allied Health Literature Plus (CINAHL Plus), and PsycInfo (OVID). Additionally, reference lists from eligible studies and systematic reviews were examined. The search was limited to English language but was not restricted by date of publication. The search strategy was developed in collaboration with an experienced librarian from the University of Birmingham, United Kingdom. The search strategy is slightly different for different databases but consistent with the concept of connection as below: (neck pain) AND (persistence OR recurrence) AND (prediction OR cohort study).

The full search strategy can be found in Data S1. ‘Persistence’ includes articles that investigated chronic neck pain and ‘recurrence’ refers to concepts including ‘repeated episode’, ‘episodic pain’ or ‘relapse’ of neck pain condition. Search terms that are related to the same concept were separated by the Boolean operator ‘OR’ and the concepts were linked by ‘AND’ for presenting the search strategy.

### 2.3 Study Selection

Duplicated articles were removed from the search results after being exported to EndNote (Clarivate Analytics, Version 20, 2020). The web‐based application Rayyan (http://rayyan.qcri.org) was used to screen the remaining articles. Two reviewers (GY and KW) independently screened the titles and abstracts of the studies based on the agreed eligibility criteria. Each study was stratified as ‘included,’ ‘excluded,’ or ‘maybe’ within the Rayyan application. A third reviewer (DF) was consulted to mediate any disagreements regarding the title and abstract selection. Full‐text screening was subsequently conducted by the same two reviewers (GY and KW) independently. In the event of conflicting interpretation, the final determination was made after consultation with the third reviewer (DF).

#### 2.3.1 Accuracy of Study Selection

Inter‐rater agreement for each stage of the study selection process was calculated using kappa statistics. Statistical analyses were conducted using IBM SPSS Statistics, Version 29.0.2023. When interpreting the kappa coefficient, a value of 0 indicates “poor” agreement, while values ranging 0–0.20 represent “slight” agreement. Values between 0.21 and 0.40 suggest “fair” agreement, 0.41–0.60 indicate “moderate” agreement, and 0.61 to 0.80 demonstrate “substantial” agreement. Scores from 0.81 to 0.99 reflect “almost perfect” agreement, with a kappa coefficient of exactly 1.00 signifying “perfect” agreement between raters (McHugh 2012).

### 2.4 Data Extraction

Data extraction was conducted by implementing a specific form created by the first reviewer (GY), which was agreed upon by the second reviewer (KW) and finalized through a preliminary trial involving three eligible studies. Both reviewers (GY and KW) conducted the data extraction then the accuracy of the extracted data was evaluated. The third reviewer (DF) was responsible for mediating any conflicts.

### 2.5 Data Items

A data extraction table was constructed with reference to the CHecklist for critical Appraisal and data extraction for systematic Reviews of prediction Modelling Studies (CHARMS) (Moons et al. 2014). The tool was initially designed for reviewing validation studies of primary prediction models but has been used with specific selection of items in certain domains for systematic reviews investigating predictive factors (Alalawi et al. 2019; Kamiya and Panlaqui 2019). The data items included: (1) authors and year of publication, (2) study design, (3) participant characteristics (age, gender), (4) sample size, (5) physical or psychological predictors, (6) outcomes of interest, (7) timepoint of measurement, (8) length of follow‐up, (9) summary of findings and (10) methods for statistical analysis.

### 2.6 Risk of Bias Assessment

The Quality in Prognosis Studies tool (QUIPS) (Hayden et al. 2013) was used to assess risk of bias of the selected studies with a standardised assessment form for each included article (Data S2). QUIPS has been modified for investigating risk of bias in prognostic studies (Hayden et al. 2006) and has acceptable inter‐rater reliability (Hayden et al. 2013). There are six key assessment domains in QUIPS: (1) study participation, (2) study attrition, (3) prognostic factor measurement, (4) confounding measurement and account, (5) outcome measurement and (6) analysis and reporting (Hayden et al. 2006). Each domain appraises the report of a specific set of information, and the report of each of these sets of information was classified as ‘yes’, ‘no’, ‘partial’ or ‘unsure’. Then, the risk of bias of each domain was categorised as ‘high,’ ‘moderate,’ or ‘low’. A domain was rated to have high risk if any rating of reporting was ‘no’ and low risk if all ratings of reporting were ‘yes’, whereas other combination of ‘yes’ with ‘partial’ or ‘unsure’ was rated moderate. A study was rated to have an overall high risk of bias if one or more domains were rated as high risk. In contrast, a study was identified to have a low overall risk of bias if all six domains were evaluated as low risk. Two reviewers (GY, KW) first independently assessed the risk of bias. Then the rating was confirmed with reference to the agreement of the two reviewers (GY, KW) (Hayden et al. 2013) and any disagreement was mediated through discussion with the third reviewer (DF).

### 2.7 Data Synthesis

Due to the heterogeneity of comparators and time assessment points, a meta‐analysis was not feasible. Studies were included for narrative and quantitative synthesis using structured tabulation and a forest plot without meta‐analysis. Narrative findings from studies were grouped based on the domain of outcomes and the domain of predictors investigated.

*Summary statistics*: The relationship between the predictor and non‐dichotomous outcome measures including NDI, pain intensity and frequency of neck pain were presented as adjusted β‐coefficient or non‐adjusted correlation coefficient (*r*) (Georgopoulos et al. 2019). The presence of a recurrent neck pain episode is a dichotomous outcome and the result was expressed as risk ratio (RR) or odd ratio (OR) from cohort studies. When RR and OR were not provided, it was estimated from the available data from the incidence of persistent or recurrent pain in the follow‐up versus the exposure and non‐exposure of predictive factors at baseline (Monaghan et al. 2021). The RR values were then converted to OR for standardisation (Grant 2014; Monaghan et al. 2021). When it was not feasible to estimate with the available data, the corresponding author of the study was contacted for raw data acquisition. The study was excluded from the forest plot when there was no response. The conversion of RR to OR followed the equation below (Grant 2014), where p0 equals the baseline risk that is the percentage incidence in the unexposed group or reference group:

$$\mathrm{RR}=\frac{\text{OR}}{\left(1-p0+\left(p0\times \text{OR}\right)\right)}$$

Forest plots without meta‐analysis were primarily categorised according to association values featured in the studies: *r*‐correlation coefficients (unadjusted correlation) and β‐coefficients (adjusted correlation). All extracted OR values were log‐transformed to β‐coefficients (Bonett 2007). When the study reported Cohen's d for standardised mean difference, it was transformed to correlation coefficient by the following equation (Rosenblad 2009).

$$r=\frac{d}{\sqrt{d^{2}+4}}$$

The confidence intervals were computed with Fisher *Z* transformation (Welz et al. 2022).

*Synthesis method*: For studies adopting multiple regression models, the result from univariable analysis of the individual predictor was extracted for constructing the forest plot. However, when the result of an individual predictor could not be isolated from the multivariable model for a forest plot analysis, only narrative analysis would be used to report the effect of the multivariable model on the recurrence or persistence of neck pain.

### 2.8 Confirmation of Predictors

The overall determination of whether a factor is predictive of persistent or recurrent neck pain was hinged on two criteria, following a previously documented approach (Alalawi et al. 2019; Kamiya and Panlaqui 2019). First, the factor must demonstrate a statistically significant effect or association with an outcome in at least 75% of the included studies. Second, the effect of the predictive factors must be reported by more than one study and must be consistently indicated with the same direction of effect across all studies.

### 2.9 Certainty of the Evidence

The Grading of Recommendations Assessment, Development and Evaluation (GRADE) tool was used to assess the certainty of the evidence (Guyatt et al. 2008). The GRADE approach has been adapted for appraising prognostic studies (Huguet et al. 2013; Iorio et al. 2015; Kamiya and Panlaqui 2019). This modified version includes two factors which can enhance the certainty of evidence: ‘moderate or large effect size’ and ‘exposure‐response gradient’ (Huguet et al. 2013). Additionally, there are six factors that can reduce the certainty of evidence: (1) phase of investigation, (2) study limitations, (3) inconsistency, (4) indirectness, (5) imprecision and (6) publication bias (Huguet et al. 2013).

The certainty of evidence for prediction of persistent and recurrent neck pain from a predictive factor was rated ‘high’, ‘moderate’, ‘low’ or ‘very low’. With reference to the GRADE Handbook (Schünemann et al. 2013), the evaluation started with the phase of investigation. When the studies reporting a predictor consisted of mainly phase 1 exploratory studies that identify potential prognostic factors which are particularly vulnerable to type I errors (false positive results), the certainty of evidence was downgraded from high to moderate as the baseline and subsequent upgrading and downgrading were conducted for the other domains (Huguet et al. 2013). In contrast, prognostic studies in phases 2 and 3 of investigation are considered to have high certainty of evidence at baseline in which a phase 2 study aims to confirm independent associations between the potential prognostic factor and the outcome with a fully developed hypothesis and a phase 3 study aims to understand a prognostic pathway (Huguet et al. 2013). For study limitations, the certainty of evidence was downgraded by one level when most of the studies had moderate risk of bias and double downgraded if the evidence was from studies of high bias for most domains according to QUIPS. For rating inconsistency, the certainty of evidence was downgraded when different studies showed inconsistent direction of results. For indirectness, the certainty of evidence was downgraded if the population, predictive factor or outcome could not represent the review question defined. For imprecision, which concerns the combined sample size of the included studies, the certainty of evidence was downgraded when there were less than ten dichotomous outcome events for each potential predictive factor or less than 100 cases at the endpoint of follow‐up for continuous outcomes. Publication bias was assumed except when contrary evidence was provided by a funnel plot and Egger test. However, the certainty of evidence would not be further downgraded if it was already downgraded in the ‘phase of investigation’ (Huguet et al. 2013). For upgrading the certainty of evidence, moderate effect size was defined as pooled r or β coefficients larger than or equal to 0.3 (Huguet et al. 2013). Lastly, an exposure‐response gradient was confirmed when an increase of effect size was proportionally associated with a change in a baseline predictive factor.

## 3 Results

### 3.1 Search Result and Study Selection

**FIGURE 1:**
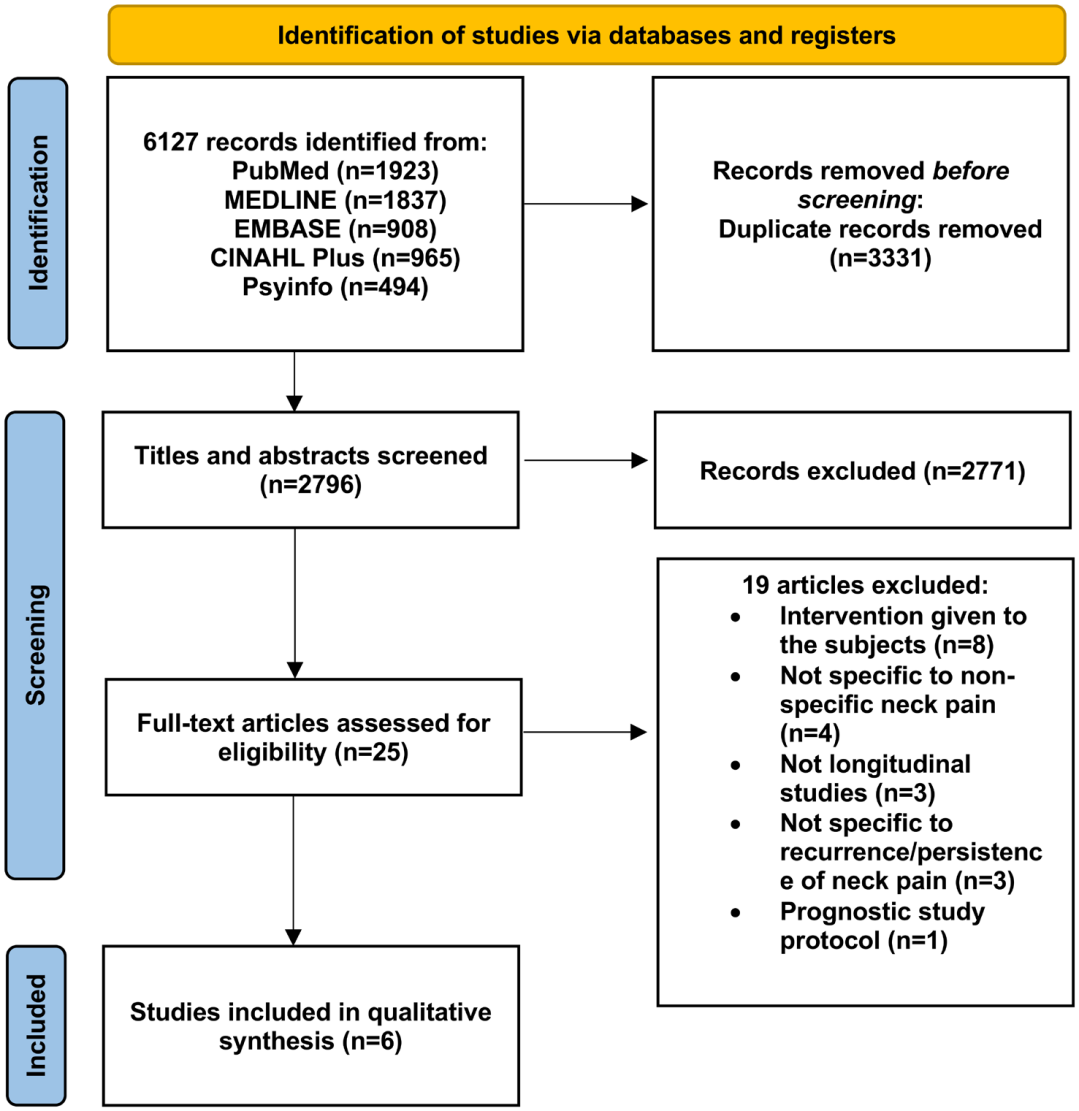
PRISMA flowchart of study selection.

The electronic search yielded 6127 records. With the removal of duplicates, 2796 titles and abstracts were screened with an almost perfect agreement of *k* = 0.99 and 25 full‐text articles were then assessed against the eligibility criteria (Figure 1). Six studies were deemed eligible for inclusion. The agreement for full‐text screening between the two reviewers was almost perfect at *k* = 0.96. The most common reason for exclusion was that active interventions were given to the cohort under investigation.

### 3.2 Study Characteristics

All six selected studies adopted a prospective cohort design (Table 1). One study investigated outcomes only on persistent neck pain (Moloney et al. 2018), three studies investigated outcomes only on recurrent neck pain (Alalawi et al. 2022; Croft et al. 2001; Xie et al. 2022), while the remaining two studies investigated both persistent and recurrent neck pain (Multanen et al. 2021; Shahidi et al. 2015). The six studies were conducted in the United Kingdom (two studies) (Alalawi et al. 2022; Croft et al. 2001), Australia (two studies) (Moloney et al. 2018; Xie et al. 2022), United States (one study) (Shahidi et al. 2015) and Finland (one study) (Multanen et al. 2021). All included studies were phase 1 prediction studies which aimed to observe association between predictors and the outcomes without testing specific hypothesis. These studies investigated a total of 2189 participants who had completed the follow‐up with the total sample comprised of 1400 (64%) females and 789 (36%) males. Most studies had a follow‐up period of 1 year except for one with 16 years (Multanen et al. 2021) and another with 6 months (Alalawi et al. 2022). Four of the six studies focused on reporting NDI (Alalawi et al. 2022; Moloney et al. 2018; Multanen et al. 2021; Shahidi et al. 2015) and one of them also reported pain intensity during the neck pain episode as a dependent variable (Moloney et al. 2018). For specifying the presence of recurrence, three of the five relevant studies reported a dichotomous outcome of the incidence of recurrence (Croft et al. 2001; Multanen et al. 2021; Shahidi et al. 2015), one study reported the number of days with recurrent pain (Alalawi et al. 2022) and one study used latent class growth analysis to stratify the outcome into two trajectories of NDI, namely Moderate‐Fluctuating Disability which indicated recurrent neck pain and another called Low‐Resolving Disability (Xie et al. 2022).

**TABLE 1:** Summary of included articles.

Authors and year of publication	Study design and targeted neck pain type of investigation	Participant characteristics	Sample size (with completion of follow up)	Physical or psychological predictors	Outcomes of interest	Timepoint of measurement of outcome	Length of follow‐up	Summary of significant or major findings	Methods for statistical analysis
Alalawi et al. (2022)	Prospective cohort; Recurrent neck pain	Recurrent neck pain during remission; Age: 31.0 ± 11.8; Female: 64%; NRS > 2 for previous neck pain episode	22	Physical: (1) ROM in flexion/extension/rotation (3) smoothness of movement in flexion/extension (4) JPE (5) submaximal CCF (7) MVC in flexion/extension Psychological: (1) TSK (2) EQ‐VAS	(1) NDI (2) Number of days with pain	(1) Baseline (predictors) (2) 6‐month follow up (outcome)	6 months	A one‐kg reduction in MVC in flexion significantly increased NDI by 0.32 units (*p* = 0.04)^a^	Least absolute shrinkage and selection operator (LASSO) regression followed by multivariable prediction model
Croft et al. (2001)	Prospective cohort; Recurrent neck pain	Healthy participants; Age 18–65; 996 females	1708	Psychological: GHQ	Report of neck pain recurrence	(1) Baseline (predictors) (2) 1‐year follow up (outcome)	1 year	The risk among those in the highest quartile of baseline GHQ score was twice as that in the lowest quartile of GHQ score (RR = 2.0)^a^	Multivariate logistic regression
Moloney et al. (2018)	Prospective cohort; Persistent neck pain	CNP; Age 42 ± 12; 30 females (76%)	51	Physical: (1) CPT (neck) (2) PPT (neck) Psychological: (1) PCS (2) DASS‐21	NDI and pain intensity in past 1 week	(1) Baseline (predictors) (2) 1‐year follow up (outcome)	1 year	Association with disability: PCS: *r* = 0.32 (*p* = 0.021)^a^ DASS‐21: *r* = 0.44 (*p =* 0.001)^a^ Association with average pain in past week: CPT: *r* = 0.29 (*p* = 0.038)^a^ PCS: 0.45 (*p* = 0.001)^a^ DASS‐21: 0.47 (*p* < 0.001)^a^	Univariate regression
Multanen et al. (2021)	Prospective cohort; Persistent and recurrent neck pain	Healthy participants; Age: 57.3 ± 11.4; All females	149	Physical: (1) Isometric strength (cervical flexion, extension and rotation) (2) ROM (sagittal, frontal and transverse planes)	NDI of recurrent or persistent pain	(1) Baseline (predictors) (2) 16‐year follow up (outcome)	16 years	Both strength and PROM did not associate with NDI (*β* < −0.1)	Univariate linear regression
Shahidi et al. (2015)	Prospective cohort; Persistent and recurrent neck pain	Healthy participants at baseline; Age: 30.2 ± 8.3: no CNP at 1 year follow up; Age: 29.8 ± 6.8: with CNP at 1 year follow up; (133 females, 34 males)	167	Physical: (1) Cervical angle (2) ROM (sagittal, frontal and transverse planes) (3) Isometric strength (cervical flexion, extension and side bending) (4) Endurance cervical flexion and extension (5) CPT (neck) (6) CPTT (neck) (7) PPT (neck) (8) CPM efficacy Psychological: (1) BDI‐II (2) STAI‐Trait score (3) PSS score (4) PCS	Persistence (≥ 3 months) or episodic recurrence (≥ 3 non‐consecutive months) with NDI ≥ 5	(1) Baseline (predictors) (2) 12 monthly questionnaires	1 year	Odd ratio: BDI‐II: 3.36^a^ Cervical extension endurance: 0.92^a^ CPM efficacy: 0.90^a^	Backward logistic regression followed by multivariate logistic regression
Xie et al. (2022)	Prospective cohort; Recurrent neck pain	Sonographer with or without neck disability; Median age 37.0 [IQR = 31.0–48.0]; Female 83.5%	92	Physical: (1) CPT (neck and TA) (2) PPT (neck and TA) (3) TS Psychological: (1) PHQ‐8 (depression) (2) GAD‐7 (anxiety) (3) PCS	Odds of being in the Moderate‐Fluctuating Disability (class 2) compared with Low‐Resolving Disability (class 1)	(1) Baseline (predictors) (2) 6‐month follow up (outcome) (3) 1‐year follow up (outcome)	1 year	Odd ratio: PHQ8: 1.47^a^ GAD‐7: 1.34^a^ PCS: 1.11^a^ CPT TA: 1.08^a^ PPT neck: 0.994^a^ PPT TA: 0.996^a^ TS neck: 1.05^a^	Latent class growth analysis and univariate logistic regression

Abbreviations: BDI‐II, Beck Depression Inventory‐II; CCF, craniocervical flexion; CNP, chronic neck pain; CPM, conditioned pain modulation; CPT, cold pain threshold; CPTT, cold pain tolerance threshold; DASS‐21, Depression Anxiety Stress Scale‐21; EQ‐VAS, European Quality of Life‐Visual Analog Scale; GAD‐7, Generalised Anxiety Disorder Scale‐7; GHQ, General Health Questionnaire; IQR, inter‐quartile range; JPE, joint position error; MVC, maximal voluntary contraction; NDI, Neck Disability Index; NRS, Numeric Rating Scale; OR, PCS, Pain Catastrophizing Scale; PHQ‐8, Patient Health Questionnaire‐8; PPT, pressure pain threshold; PSS, Perceived Stress Scale; ROM, neck range of motion; RR, risk ratio; STAI‐Trait, Spielberger State–Trait Inventory–Trait index; TA, tibialis anterior; TS, temporal summation; TSK, Tampa Scale of Kinesiophobia.

^a^ Statistically significant findings.

### 3.3 Risk of Bias

Three studies were rated to have high overall risk of bias and the other three were rated with moderate overall risk of bias (Table 2). While all studies were able to sufficiently report outcome measurements and demonstrate low risk in analysis and reporting, moderate to high risk of bias was evident for the domain of ‘study confounding’, which means risk of bias could be minimised if the studies reported major confounding factors in detail with adjustment done in the subsequent regression analysis. High risk of bias for study population was noticed in one study as the population consisted of a mix of patients with NSNP and whiplash associated disorder and the report of results for the two conditions was not separated (Moloney et al. 2018). Furthermore, high risk of bias for reporting attrition was noticed in one study with lack of information on the characteristics of the dropouts for the follow‐up (Multanen et al. 2021).

**TABLE 2:** Risk of bias assessment.

Study	Study participation	Study attrition	Prognostic factor measurement	Outcome measurement	Study confounding	Analysis and reporting	Overall risk of bias
Alalawi et al. (2022)	Low	Moderate	Moderate	Low	Moderate	Low	**Moderate**
Croft et al. (2001)	Low	Moderate	Moderate	Low	Moderate	Low	**Moderate**
Moloney et al. (2018)	High	Low	Low	Low	High	Low	**High**
Multanen et al. (2021)	Low	High	Moderate	Low	Moderate	Low	**High**
Shahidi et al. (2015)	Low	Low	Moderate	Low	Moderate	Low	**Moderate**
Xie et al. (2022)	Low	Low	Moderate	Low	High	Low	**High**

### 3.4 Predictors Identified: A Narrative Synthesis

**FIGURE 2:**
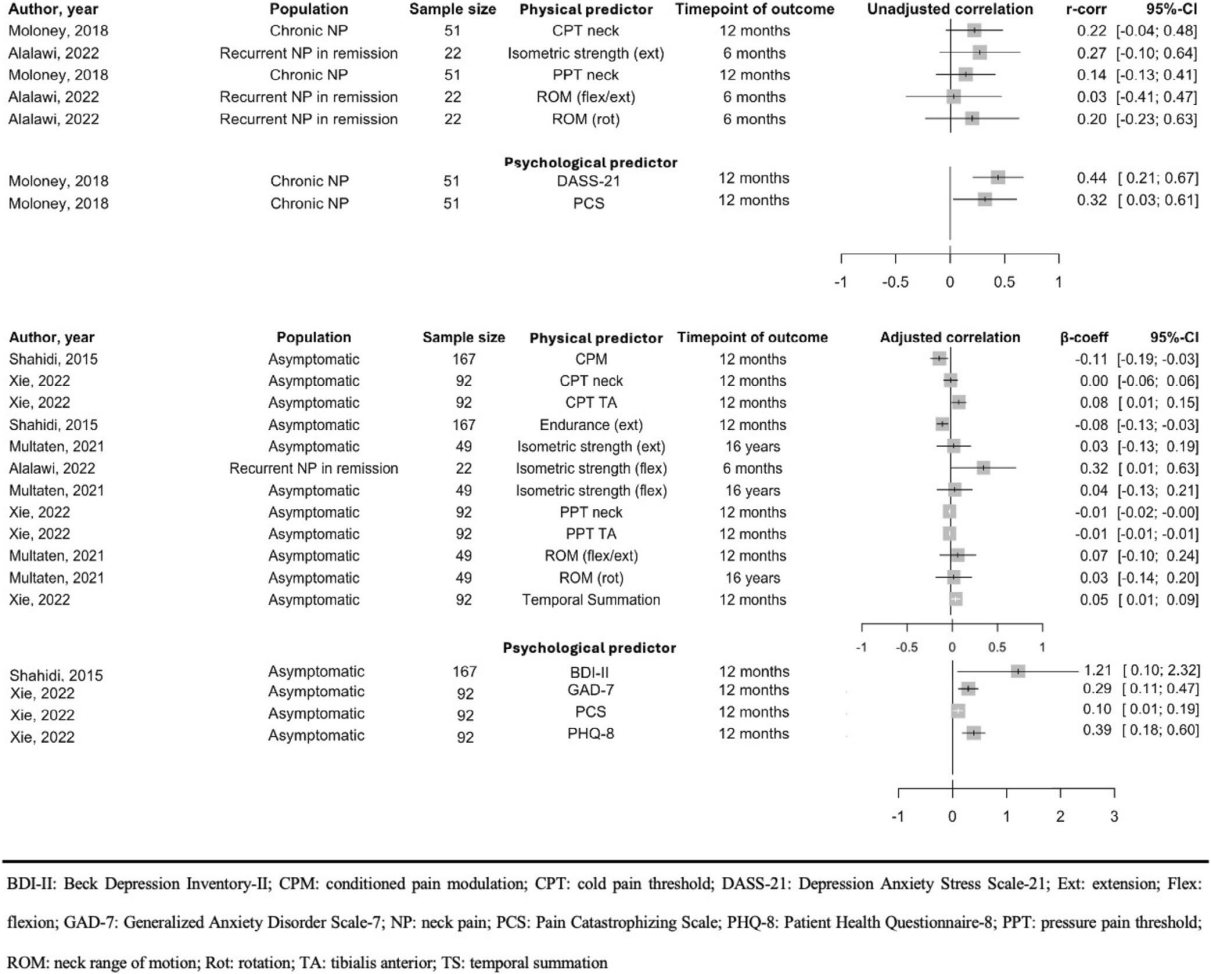
Forest plots, without meta‐analysis, describing the overall association (r‐correlations and β‐coefficients) between predictors and follow‐up neck disability.

**FIGURE 3:**
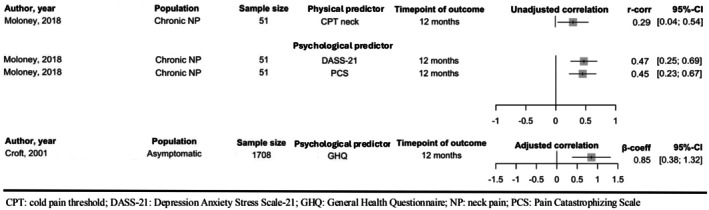
Forest plots, without meta‐analysis, describing the overall association (r‐correlations and β‐coefficients) between predictors and follow‐up neck pain intensity and incidence.

Nine predictors were reported to have a significant association with neck disability and three predictors were reported as having significant association with pain intensity and incidence. The certainty of evidence of significant predictors investigated by at least one study and non‐significant predictors investigated in more than one study were appraised using GRADE (Tables 3 and 4). The physical predictors covered a wide range of neuromuscular and sensory features including cervical kinematics, modified muscle activity and sensory function. As no predictors were reported by at least five studies, a narrative analysis was adopted. For visualising the results, Figures 2 and 3 present forest plots without meta‐analysis for the results on predictors with significant association or which were reported in more than one study and when the summary statistics were same (*r*‐correlations or β‐coefficients).

**TABLE 3:** Overall certainty of evidence—predictors of persistent or recurrent neck disability.

Potential predictor identified	Number of participants	Number of studies	Univariate			Multivariate			Phase	GRADE factors	Inconsistency	Indirectness	Imprecision	Publication bias	Moderate/large effect size	Exposure‐response gradient	Overall certainty
			+	0	−	+	0	−		Study limitations							
Neck range of motion	238	3	0	1	0	0	2	0	1	x	✓	✓	✓	✓	x	NA	++
Isometric strength (flexion)	238	3	0	1	0	0	1	1	1	x	x	✓	✓	✓	x	NA	+
Isometric strength (extension)	238	3	0	1	0	0	2	0	1	x	✓	✓	✓	✓	x	NA	++
Cold pain threshold (neck)	310	3	0	2	0	0	1	0	1	x	✓	✓	✓	✓	x	NA	++
Cold pain threshold (tibialis anterior)	92	1	1	0	0		NA		1	x	NA	✓	✓	✓	x	NA	++
Pressure pain threshold (neck)	310	3	0	1	1	0	1	0	1	x	x	✓	✓	✓	x	NA	+
Pressure pain threshold (tibialis anterior)	92	1	0	0	1		NA		1	x	NA	✓	✓	✓	x	NA	++
Conditioned pain modulation	167	1		NA		0	0	1	1	x	NA	✓	✓	✓	x	NA	++
Endurance (extension)	167	1		NA		0	0	1	1	x	NA	✓	✓	✓	x	NA	++
Temporal summation	92	1	1	0	0		NA		1	x	NA	✓	✓	✓	x	NA	++
Pain Catastrophizing Scale	143	2	2	0	0		NA		1	x	✓	✓	✓	✓	x	NA	++
Psychological distress^a^	310	3	2	0	0	1	0	0	1	x	✓	✓	✓	✓	✓	NA	+++

*Note:* For uni‐ and multivariate analyses: + represents number of significant effects with a positive association; 0 represents number of non‐significant effects; − represents number of significant effects with a negative association. Phase means phase of investigation. For GRADE factors: ✓, no serious limitations; ✕, serious limitations (or ‘not present’ for moderate/large effect size, dose effect); NA, unable to rate item based on available information. For overall certainty of evidence: + very low; ++ low; +++ moderate; ++++ high. Only factors investigated in more than one study or which showed statistical significance in at least one study are included in the table.

^a^ Psychological distress involves findings from DASS‐21, BDI‐II, PHQ‐8 and GAD‐7.

**TABLE 4:** Overall certainty of evidence—predictors of persistent or recurrent neck pain intensity and incidence.

Potential predictor identified	Number of participants	Number of studies	Univariate			Multivariate			Phase	GRADE factors	Inconsistency	Indirectness	Imprecision	Publication bias	Moderate/large effect size	Exposure‐response gradient	Overall certainty
			+	0	−	+	0	−		Study limitations							
Cold pain threshold (neck)	51	1	1	0	0	0	0	0	1	x	NA	✓	✓	✓	x	NA	++
Pain Catastrophizing Scale	51	1	1	0	0	0	0	0	1	x	NA	✓	✓	✓	✓	NA	+++
Psychological distress^a^	1759	2	1	0	0	1	0	0	1	x	✓	✓	✓	✓	✓	NA	+++

*Note:* For uni‐ and multivariate analyses: + represents number of significant effects with a positive association; 0 represents number of non‐significant effects; − represents number of significant effects with a negative association. Phase means phase of investigation. For GRADE factors: ✓, no serious limitations; ✕, serious limitations (or ‘not present’ for moderate/large effect size, dose effect); NA, unable to rate item based on available information. For overall certainty of evidence: + very low; ++ low; +++ moderate; ++++ high. Only factors investigated in more than one study or which showed statistical significance in at least one study are included in the table.

^a^ Psychological distress involves findings from GHQ and DASS‐21.

### 3.5 Predicting Neck Disability

Seven physical predictors were reported to have significant association with persistent and recurrent neck disability; these included isometric neck flexion strength (Alalawi et al. 2022), cold pain threshold over the tibialis anterior (CPT TA) (Xie et al. 2022), pressure pain threshold over the neck (PPT neck) (Xie et al. 2022), pressure pain threshold over the tibialis anterior (PPT TA) (Xie et al. 2022), conditioned pain modulation (CPM) (Shahidi et al. 2015), neck extensor endurance (Shahidi et al. 2015) and temporal summation (TS) (Xie et al. 2022). Although investigated by multiple studies, the certainty of evidence for isometric neck flexion strength (Alalawi et al. 2022; Multanen et al. 2021; Shahidi et al. 2015) and PPT measured over the neck (Moloney et al. 2018; Shahidi et al. 2015; Xie et al. 2022) was affected by the phase of investigation, study limitation, inconsistency and a lack of moderate to large effect size. Investigated in only one study, CPT TA (Xie et al. 2022) and TS (Xie et al. 2022) showed a significant positive association with neck disability, while PPT TA (Xie et al. 2022), CPM (Shahidi et al. 2015) and neck extensor endurance (Shahidi et al. 2015) showed a negative association. More than one study consistently reported that cervical range of motion (ROM) (Alalawi et al. 2022; Multanen et al. 2021; Shahidi et al. 2015), cold pain threshold measured over the neck (CPT neck) (Moloney et al. 2018; Shahidi et al. 2015; Xie et al. 2022) and isometric neck extension strength (Alalawi et al. 2022; Multanen et al. 2021; Shahidi et al. 2015) were not associated with persistent and recurrent neck disability. Overall, evidence to support that isometric neck flexion strength, and PPT over the neck as predictors of persistent and recurrent neck disability was contrasting, and no meaningful conclusion can be drawn. With only a single study available for each, no meaningful conclusions can be drawn for CPT TA, PPT TA, neck extensor endurance, TS, and CPM even they showed significant association. Low certainty of evidence showed that neck ROM, CPT over the neck and isometric neck extension strength are not associated with persistent and recurrent neck disability.

Since depression, anxiety and stress were often assessed together with different questionnaires across studies, results were grouped and presented together referring to ‘psychological distress’. With this definition, two psychological predictors were reported in the included studies which showed significant association with persistent and recurrent neck disability. These predictors are the pain catastrophizing scale (PCS) and psychological distress which was reflected by four psychological assessment tools including the Depression, Anxiety and Stress Scale‐21 (DASS‐21), Beck Depression Inventory‐II (BDI‐II), Patient Health Questionnaire‐8 (PHQ‐8) and General Anxiety and Depression Scale‐7 (GAD‐7). PCS was not considered as a measure of psychological distress. It was reported by two studies which consistently showed its positive association with neck disability (Moloney et al. 2018; Xie et al. 2022). Similarly, DASS‐21 (Moloney et al. 2018), BDI‐II (Shahidi et al. 2015), PHQ‐8 (Xie et al. 2022) and GAD‐7 (Xie et al. 2022) showed positive and significant associations with neck disability. The certainty of evidence for both pain catastrophizing and psychological distress was downgraded because evidence only came from phase 1 studies with study limitation on risk of bias. As studies investigating psychological distress consistently reported at least a moderate effect size (*β* or *r* > 0.3), the certainty of evidence was rated moderate. In contrast, the rating for pain catastrophizing as a predictor of future neck disability remained at low certainty of evidence because one of the two studies reported a small effect size (Xie et al. 2022).

### 3.6 Predicting Neck Pain Intensity and Incidence

For physical predictors, CPT over the neck was positively associated with the intensity of persistent neck pain (Moloney et al. 2018). However, this was investigated in only one study and no meaningful conclusions can be drawn.

For psychological predictors, PCS (Moloney et al. 2018) and psychological distress were reported to have significant positive association with persistent or recurrent neck pain intensity and incidence, and psychological distress was assessed in two studies using DASS‐21 (Moloney et al. 2018) and General Health Questionnaire (GHQ) (Croft et al. 2001). The certainty of evidence was downgraded because both studies were phase 1 studies with study limitation on risk of bias. As all studies reported at least a moderate effect size, moderate certainty of evidence supports that PCS and psychological distress are associated with persistent and recurrent neck pain intensity and incidence.

## 4 Discussion

This systematic review investigated studies that assessed physical and psychological factors and their association with persistent and recurrent neck pain and disability. Low to moderate certainty of evidence was found in support that high pain catastrophizing and psychological distress (including depression, anxiety and stress) may predict persistent and recurrent neck disability, pain intensity and incidence. For physical factors, because of the inconsistency of results and the limited number of studies available, further studies are needed to confirm an association between isometric neck flexion strength, CPT TA, PPT neck, PPT TA, CPM, neck extensor endurance, and TS with persistent and recurrent neck disability and pain.

The significant findings that pain catastrophizing and psychological distress (including depression, anxiety and stress) are predictors of neck pain align with previous studies on chronic spinal pain. A systematic review reported that depression is related to LBP disability and pain with an OR that ranged 1.04–2.47 (Pinheiro et al. 2016). Catastrophizing of pain (Swinkels‐Meewisse et al. 2006) was reported to be positively associated with perceived disability in LBP, while anxiety, depression (Wenzel et al. 2002) and the PCS score were positively associated with pain intensity in people with whiplash‐associated disorders (Andersen et al. 2016). These convergent results support the implementation of screening for pain catastrophizing and psychological distress in the early stage of neck pain management to identify patients who are likely to develop persistent or recurrent neck pain. A recent systematic review revealed that psychological interventions including cognitive behavioural therapy delivered by physiotherapists in addition to standard treatment are more effective than standard treatment alone in reducing neck pain and disability (Farrell et al. 2023). Such psychological interventions target maladaptive beliefs and negative thoughts (Monticone et al. 2012). Therefore, psychological interventions with education to reduce pain‐related distress and catastrophizing should be incorporated into the management of patients with high pain catastrophizing beliefs and psychological distress to prevent long‐lasting neck pain and disability.

There was inconsistency among the studies reporting the association of PPT measured over the neck with persistent and recurrent neck disability (Moloney et al. 2018; Shahidi et al. 2015; Xie et al. 2022). All of the involved studies measured PPT over the neck specifically over cervical facet joints or over the upper trapezius. A recent systematic review and meta‐analysis indicated lower PPT over the neck in people with chronic neck pain (Nunes et al. 2021). Although the findings in our study do not support PPT over the neck region as a predictor of persistent or recurrent NP, it should be considered that individuals with higher scores in pain‐related questionnaires and with higher scores of central sensitisation tend to present with lower PPT values (Suzuki et al. 2022). It is speculated that if there was subgroup analysis of patients with these presentations, then the value of PPT over the neck as a predictor may be stronger.

The report of isometric neck flexion strength as a predictor of future neck disability showed inconsistency between the three studies included (Alalawi et al. 2022; Multanen et al. 2021; Shahidi et al. 2015); this inconsistency can at least be partially explained by the heterogeneity of baseline neck pain history. The only study that reported a significant association of decreased neck flexion strength with increased future neck disability assessed the baseline neck flexion strength of participants during a period of remission from neck pain. This study also provided a clear definition of remission such that these participants had to have had at least two recent episodes of neck pain in the prior 12 months with clinically significant pain (NPRS ≥ 2/10) and disability (NDI ≥ 10/50). It is important to note that a history of neck symptoms has been reported to be associated with greater future neck disability (Alalawi et al. 2022; Schellingerhout et al. 2010). In contrast, the other two studies that reported no association between baseline neck flexion strength and future neck disability examined asymptomatic participants in the initial assessment and did not specify if they had any recent history of neck symptoms (Multanen et al. 2021; Shahidi et al. 2015). This difference in baseline neck pain history can therefore be a factor contributing to the different association levels reported in the three studies.

Three studies reported no association between cervical ROM and persistent and recurrent neck pain (Alalawi et al. 2022; Multanen et al. 2021; Shahidi et al. 2015). Nevertheless, the certainty of evidence was rated low. In particular, two studies had small sample sizes which led to insufficient statistical power (Alalawi et al. 2022; Multanen et al. 2021). It is important to note that the average values for ROM for the participants in all three studies indicate that in general, the participants did not have impaired ROM at baseline when compared with normative values (Thoomes‐de Graaf et al. 2020) or data from healthy controls in the experiment. Therefore, the conclusion that ROM has no predictive value may not be justified as a predictor should be an impairment in order to be relevant. From a clinical perspective, reduced ROM is a common functional limitation prompting intervention. Therefore, addressing ROM impairment when present remains clinically relevant to improve neck function. It should also be noted that it may not be appropriate to overinterpret that CPT over the neck region and isometric extension strength have no association with persistent and recurrent neck pain, as there are no normative data established for CPT measured over the neck region and one of the three studies assessing isometric extension strength investigated healthy individuals who had better strength than normative reference values during baseline measurement (Catenaccio et al. 2017; Multanen et al. 2021).

### 4.1 Methodological Considerations

There are some limitations that need to be acknowledged. The overall certainty of evidence for all physical predictors ranged from very low to low. The certainty was mainly downgraded because of the phase of investigation and the domain of ‘study limitation’ which reflected moderate to high risk of bias as evaluated by QUIPS. For the phase of investigation, prospective or retrospective cohort studies that evaluate a well‐defined hypothesis and conceptual framework, free from significant study limitations, along with confirmatory studies without major flaws, are those that provide high‐quality evidence regarding prediction. Therefore, it is these types of studies (phase 2 or 3) that should be prioritised in future research (Huguet et al. 2013). Furthermore, for the investigation of the population that was pain free at baseline, only one study specified that the group was in remission of neck pain. This means there could be a heterogeneity of the included population among studies such that the ‘healthy’ participants might comprise patients who had recurrent neck pain before and could manifest with a different likelihood to develop future symptoms.

### 4.2 Implications

The findings from this systematic review indicate that high pain catastrophizing and psychological distress may predict persistent and recurrent neck pain and disability. High‐quality prediction studies with low risk of bias and precise directional hypotheses between predictors and neck pain outcomes are needed in the future to improve the certainty of evidence. In addition, future prospective cohort studies should investigate other important potential predictors that have been shown to be altered in patients with NSNP. Future investigations may explore common physical factors associated with neck pain including but not limited to neck muscle coactivation (Tsang et al. 2014), force steadiness (Muceli et al. 2011), delayed onset of neck muscle activity (Falla et al. 2004) and brain structural changes (Chaikla et al. 2025).

With regard to clinical implications, the addition of early assessment of pain catastrophizing and psychological distress (including depression, anxiety and stress) is advocated to identify patients that are more susceptible to persistent and recurrent neck pain. When warranted, psychological interventions targeting patients' maladaptive beliefs, pain‐related anxiety and catastrophizing should be considered within the rehabilitation programme to minimise the likelihood of persistent and recurrent neck pain and disability.

## 5 Conclusion

This systematic review investigated predictors for persistent and recurrent neck disability, pain intensity and incidence from six prospective cohort studies. The results indicated that only high pain catastrophizing and psychological distress (including depression, anxiety and stress) were reported consistently to positively associate with persistent and recurrent neck pain and disability. In relation to physical factors, because of the inconsistency of results and limited number of studies available, further studies are needed to confirm findings of an association between isometric neck flexion strength, CPT TA, PPT neck and PPT TA, CPM, neck extensor endurance, and TS with persistent neck pain and disability.

## Author Contributions

All authors contributed to the development of the systematic review. C.W.G.Y., K.W. and D.F. are the first, second and third reviewers. C.W.G.Y. and K.W. are PhD students with D.F. as Lead Supervisor and J.A.D. and M.M. as Co‐Supervisors. C.W.G.Y. constructed the review with guidance from D.F., J.A.D., M.M., K.W. and V.D. All authors provided feedback on the preparation of the manuscript, and all authors approved the final version for publication. D.F. is the guarantor.

## Conflicts of Interest

The authors declare no conflicts of interest.

## Supporting information


**Table S1:** Concepts and keywords.
**Table S2:** Search strategy by database.

**Table S3:** Assessment form of Quality in Prognosis Studies tool (QUIPS) for individual article.
