# Supplementary material for: Physical and Psychological Predictors for Persistent and Recurrent Non‐Specific Neck Pain: A Systematic Review
Source: Eur J Pain. 2025 Nov 12;29(10):e70168. doi: 10.1002/ejp.70168 (PMC12611179; doi:10.1002/ejp.70168)
Supplement: Supplementary file 1 — Table S1: Concepts and keywords. Table S2: Search strategy by database. [file EJP-29-0-s002.pdf]

Research question: Can physical and psychological factors predict recurrent or persistent non-specific neck pain and disability?

Table S1. Concepts and keywords

Concepts	Keywords
Neck pain	Cervical pain OR neck ache OR cervicalgia
Persistence	Persist* OR chronic*
Recurrence	Recurr* OR repeat* OR relapse OR episodic
Prediction	Predict* OR prognos* OR risk factor OR associat*

Table S2. Search strategy by database

Database / Search strategy
**PubMed** ('neck pain' [MeSH]) AND (('persist*' OR 'chronic') OR ('recurr*' OR 'relapse' OR ‘repeat*’ OR ‘remission’ OR ‘episod*’)) AND (('predict*' OR 'prognos*' OR 'risk factor*' OR ‘associat*’) OR ('longitudinal' OR 'cohort' OR 'prospective' OR 'follow up'))
**MEDLINE** (Exp 'neck pain') AND (('persist*' OR 'chronic neck pain').mp OR ('recurr*' OR 'relapse' OR ‘repeat*’ OR ‘remission’ OR ‘episod*’).mp) AND (('predict*' OR 'prognos*' OR 'risk factor*' OR ‘associat*’).mp OR ('longitudinal' OR 'cohort' OR 'prospective' OR 'follow up').mp)
**EMBASE** (Exp 'neck pain') AND (('persist*' OR 'chronic neck pain').mp OR ('recurr*' OR 'relapse' OR ‘repeat*’ OR ‘remission’ OR ‘episod*’).mp) AND (('predict*' OR 'prognos*' OR 'risk factor*' OR ‘associat*’).mp OR ('longitudinal' OR 'cohort' OR 'prospective' OR 'follow up').mp)
**CINAHL Plus** (MH ‘neck pain’) AND (('persist*' OR 'chronic') OR ('recurr*' OR 'relapse' OR ‘repeat*’ OR ‘remission’ OR ‘episod*’)) AND (('predict*' OR 'prognos*' OR 'risk factor*' OR ‘associat*’) OR ('longitudinal' OR 'cohort' OR 'prospective' OR 'follow up'))
**Psycinfo** ('neck pain' .mp) AND (('persist*' OR 'chronic neck pain').mp OR ('recurr*' OR 'relapse' OR repeat* OR remission OR episod*).mp) AND (('predict*' OR 'prognos*' OR 'risk factor*' OR ‘associat*’).mp OR ('longitudinal' OR 'cohort' OR 'prospective' OR 'follow up').mp)
